# Exploring mechanisms that explain how coalition groups are formed and how they work to sustain political priority for maternal and child health in Nigeria using the advocacy coalition framework

**DOI:** 10.1186/s12961-020-00660-3

**Published:** 2021-03-01

**Authors:** Chinyere Okeke, Ana Manzano, Uche Obi, Enyi Etiaba, Obinna Onwujekwe, Tolib Mirzoev, Benjamin Uzochukwu

**Affiliations:** 1 Department of Community Medicine, College of Medicine, University of Nigeria Enugu Campus, Enugu, Nigeria; 2 School of Sociology and Social Policy, University of Leeds, Leeds, United Kingdom; 3 Department of Community Medicine, University of Nigeria Teaching Hospital Enugu, Enugu, Nigeria; 4 Department of Health Administration and Management College of Medicine, University of Nigeria Enugu Campus, Enugu, Nigeria; 5 Nuffield Centre for International Health and Development, University of Leeds, Worsley Building, Clarendon Way, Leeds, United Kingdom

**Keywords:** Advocacy coalition, Advocacy coalition framework, MCH, Policy Nigeria

## Abstract

**Background:**

The unacceptably high rate of maternal and child mortality in Nigeria prompted the government to introduce a free maternal and child health (MCH) programme, which was stopped abruptly following a change in government. This triggered increased advocacy for sustaining MCH as a political priority in the country and led to the formation of advocacy coalitions. This study set out to explain the process involved in the formation of advocacy coalition groups and how they work to bring about sustained political prioritization for MCH in Nigeria. It will contribute to the understanding of the Nigerian MCH sector subsystem and will be beneficial to health policy advocates and public health researchers in Nigeria.

**Methods:**

This study employed a qualitative case study approach. Data were collected using a pretested interview guide to conduct 22 in-depth interviews, while advocacy events were reviewed pro forma. The document review was analysed using the manual content analysis method, while qualitative data audiotapes were transcribed verbatim, anonymized, double-coded in MS Word using colour-coded highlights and analysed using manual thematic and framework analysis guided by the advocacy coalition framework (ACF). The ACF was used to identify the policy subsystem including the actors, their belief, coordination and resources, as well as the effects of advocacy groups on policy change. Ethics and consent approval were obtained for the study.

**Results:**

The policy subsystem identified the actors and characterized the coalitions, and described their group formation processes and resources/strategies for engagement. The perceived deep core belief driving the MCH agenda is the right of an individual to health. The effects of advocacy groups on policy change were identified, along with the factors that enabled effectiveness, as well as constraints to coalition formation. External factors and triggers of coalition formation were identified to include high maternal mortality and withdrawal of the free MCH programme, while the contextual issues were the health system issues and the socioeconomic factors affecting the country.

**Conclusion:**

Our findings add to an increasing body of evidence that the use of ACF is beneficial in exploring how advocacy coalitions are formed and in identifying the effects of advocacy groups on policy change.

## Introduction/background

Maternal and child mortality remain unacceptably high in Nigeria and have yet to be adequately addressed. Despite a global reduction in maternal and child mortality (with a 44% reduction in maternal mortality and a 53% decline in child mortality [1]), rates in Nigeria remains high, with recorded mortality rates of 576/100 000 live births in 2013 [2] to 814/100 000 live births in 2015 [1] and 512/100 000 live births in 2018 [3]. Given that the United Nations Sustainable Development Goals (SDGs) aim to reduce the global maternal mortality to less than 70 per 100 000 live births and under-5 mortality to 25 per 1000 live births in all countries [4], Nigeria runs a very high risk of being left behind.

Persistent high maternal and child mortality has been attributed to high levels of poverty, inconsistencies in policy, policy reversals, corruption, the weak implementation of strategies, and general environmental uncertainty [5, 6]. To help address this, the government in 2012 launched the maternal and child health (MCH) component of the Subsidy Reinvestment and Empowerment (SURE-P) programme to invest the gains from the fuel subsidy into a social protection scheme. This programme had a supply side and a demand side component. The supply side component aimed to broaden access to maternity services and improve health outcomes through infrastructural upgrade, supply of medical and surgical consumables, and increased number of midwives, community health extension workers and village health workers, while the demand side component provided conditional cash transfers to pregnant women. However, the programme was stopped abruptly with a change in government, and this caused great concern about the welfare of mothers and children who initially received free care and financial incentives to access services [7].

The SURE-P MCH programme ended abruptly in 2015, with the change in the political tenure of the government that instituted it. This was accompanied by sudden withdrawal of associated funds without prior notice, resulting in no alternative for free health care for mothers and children. This was of great concern to most citizens of the country and led many individuals and groups to take action to maintain MCH as a political priority in the country. They recognized the need for MCH to gain political commitment, policy support, social acceptance and systems support, which was described by the World Health Organization (WHO) in 1995 to mean “advocacy” [8]. Advocacy coalitions play an increasingly prominent role within the national health landscape, linking actors and institutions to attract political attention and resources for subjects of interest [9].

The advocacy coalition concept was described by Sabatier and Jenkins as a collection of people from different public and private institutions at various tiers of government who have similar beliefs and aspirations, and set out to influence policies, budgets and government decision-makers to achieve their aspirations over time [10]. These people are called advocates, and they strive to bring about a change in the policy process that will have a greater positive effect on a large number of people than individual services and programmes alone can achieve [11]. There are different perspectives on modalities for effective advocacy. Some scholars argue that advocacy is most effective when members frame the issue of interest such that it includes a collective understanding of the problem (in this case, the poor MCH indices), with strong reason to advocate and agreement on expected outcomes. This is followed by the formation of a coalition made up of individuals and groups from various areas of the health sector, with full involvement in the politics of the problem and not just its technical component. The MCH advocates saw a need to form coalitions and join resources in the hope of having their voices heard and to achieve a greater impact than would be possible for them individually. Coalition-building is not an easy process, but when coalitions are well conceived and managed, they are of great benefit to members. These benefits include shared goals and commitments, successful networking and sharing of information, improved access to resources, heightened accountability, and improved problem-solving [12].

This study uses a political science concept to inform the study of public health through the advocacy coalition framework (ACF). It has been acknowledged as one of the foremost theoretical frameworks for analysing policy processes [13, 14, 15] as it offers a comprehensive framework for thinking about political engagement [16]. The ACF was developed to simplify the complexity of public policy processes [17], thus its selection for this study. Advocacy coalitions are defined by their shared beliefs and coordinated actions which are the drivers of policy change [18].

ACF has been used mostly in sector reform evaluations, with little application in developing countries [19]. The importance of advocacy coalition interventions for policy agenda setting, formulation, implementation and evaluation has thus far been less regarded, despite its being practised for a long time. Sabatier and Jenkins identified that the major hindrance to its applicability outside of the United States and Europe was the lack of expansive sets of actors that needed to be involved in policy-making, the weakness of the civil society, and lack of technical expertise. However, the analysis of this framework has illustrated its relevance in public health as used in different studies, and in different settings [18, 20, 21, 22].

This article uses three core parts of the ACF, namely policy subsystem, external events and core beliefs, in explaining the mechanism of advocacy coalition for MCH in Nigeria, in order to arrive at some conclusions of political engagements that will be useful to public health advocates and researchers. Researchers studying coalitions often seek answers to questions pertaining to their formation, their beliefs and coordination. These were also explored in this study.

There are other theories proposed for the study of group activities in the achievement of set goals, such as the social movement theory, which provides an understanding of group formation, strategies and persistence but does not explain policy change [23], and likewise the group formation theory [24], which deals with the various stages a group will undergo for them to harmonize and be able to work together. Though some of the characteristics may be applicable to advocacy coalition groups, it does not really portray the political interplay and the ability to bring about a policy change possible through the ACF. There is also the community organizing theory [25], which is used to explain issues of increased empowerment and community competence, which is not applicable in this study.

This study uses ACF to explain advocacy coalition formation and how it works to bring about a sustained political priority for MCH in Nigeria, following the withdrawal of the free MCH programme. There is limited evidence specific to low- and middle-income country (LMIC) contexts on the use of ACF. This article will then contribute towards advancing ACF research in Africa and further show the flexibility and wide applicability of the framework in understanding public health issues. This is appropriate given the limited research conducted on understanding advocacy coalition groups in LMICs. It will answer the research question: How are advocacy coalitions formed and how did they contribute to prioritization of the Maternal Newborn and Child Health (MNCH) programme in Nigeria? This paper will confirm or refute one of the traditional ACF hypotheses about coalition which states that actors within an advocacy coalition will show substantial consensus on issues pertaining to the policy core. This study will also contribute to the understanding of the Nigerian health sector subsystem, especially that of the MCH policy environment in explaining what motivated the formation of advocacy coalitions and how they function in maintaining MCH on the political agenda in Nigeria. This study will benefit health policy advocates and public health researchers in Nigeria.

The theoretical underpinning of this research work derives from the fact that ACFs have been used recently in several contexts to identify coalitions [26, 27, 28]. Most studies follow a four-step approach for identifying coalitions, by first defining the boundaries of the subsystem, then identifying the policy actors and potential coalition members, then measuring the different beliefs of those actors in deductive, inductive or explorative ways, and finally, investigating theoretically relevant characteristics of coalitions, such as coordination networks across coalition actors, as stated in the collection of ACF applications in Weible et al. (2016). Others have added resources to this as well [13]. This study also adopts some of these methods but did not search systematically for evidence of coordination among all the actors in the subsystem. However, it used the ACF to identify advocacy coalition groups and then looked at how they worked to sustain MCH issues as a priority in the country. It is known that ACF has provided the basis for successful qualitative method approaches as used in the past [29], and so it was adopted for this study.

### Conceptual framework

**Fig. 1:**
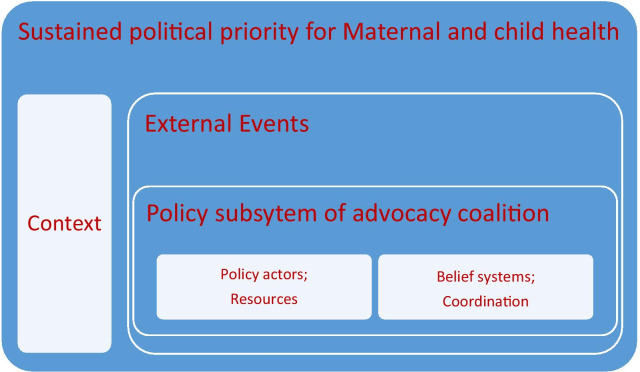
Conceptual framework of the formation of advocacy coalitions and how they work to sustain political priority for maternal and child health in Nigeria

This study was adapted and guided by the Sabatier and Weible ACF (2007) [13]. The ACF here is about individuals, their collective interactions, the external factors and the context in which it was applied. This study looks at MCH advocacy coalition formation and how it worked to sustain MCH priority in Nigeria. It assumes that policy development occurs within policy subsystems, so understanding the political dynamics of policy subsystems is essential to explaining policy change. Within this subsystem are the various actors from both public and private organizations who are actively involved in seeking solutions to a policy problem. Usually they are those who are acquainted with the issues involved and have been, over time, actively participating or have sought to participate in policy formulations in the domain. In the ACF, actors in policy subsystems are fundamentally driven by their beliefs and their desire to see their beliefs reflected in policy. These could be deep core beliefs, policy core beliefs or secondary beliefs, otherwise known as the interests of members of a group. At the same time, the subsystem is affected by external factors which, according to Sabatier and Weible [14], could be a relatively stable parameter, or an external shock. This is a necessary catalyst of major policy change, as it provides a stimulus to change that which is totally outside the control of the subsystem. Those external events are important because they not only shock the policy subsystem, but also shift public attention towards the subsystem (Fig. 1). The institutional context (i.e. formal and informal norms that facilitate or limit political actors’ behaviour) influences the formation of coalitions, the stability and maintenance of the coalition, and the strategies used and resources available to them.

## Methods

### Research design

This is part of a larger study that sought to determine the effectiveness of a novel community health workers programme in improving MCH in Nigeria [30]. A qualitative case study approach [31] was employed, which is appropriate for studies seeking to understand complex issues, giving meaning to the experiences of the participants in the setting it occurred. A case study approach has the advantage of depth and a focus on obtaining a rich complete picture. In this study, advocacy groups for MCH were the cases and thus the “units of analysis”. The mechanism of how the groups were formed, how they function and the roles they play to keep and sustain MCH high on the policy agenda within the country was explored in depth at the national and subnational levels. This allowed conclusions to be drawn about these advocacy groups within their specific contexts.

### Study setting, sampling and selection of research participants

The study was carried out in Nigeria, which has a federal democratic political structure, with a Federal Capital Territory (FCT) based in Abuja and 36 autonomous states. The country has two main political parties, the People’s Democratic Party (PDP) and the All Progressives Congress (APC). The PDP suffered a major defeat in Nigeria’s 2015 elections and lost the presidency, the house, the senate and most state governorships for the first time since the 1999 transition to democracy. The political succession occurs every four years. Unfortunately, in Nigeria, the political checks and balances are not effective, as real power and control resides almost exclusively with the executive arm of government. The legislative arm and the judiciary are seen to be always subservient to the executive.

The national health policy in Nigeria was developed in 1988, and revised in 2004 and 2016. However, the various aspects of the health sector develop their policies and programmes in accordance with identified needs, and measures to address them.

The writers are researchers in academia and have no political interests, but are health systems and policy researchers with interest in MCH among other disciplines.

### Document review/mapping of advocacy events

A listing of advocacy activities carried out post SURE-P was obtained by mapping of policies, programmes and advocacy events (the purpose was to map changes in policy and programme environments at the federal and state levels as well as mapping advocacy and lobbying events that helped to keep MCH on the political agenda), and suitable participants from the identified activities were invited to participate in the study. The document review included a systematic search in published peer-reviewed literature including project and agency reports, news stories and articles published as a result of advocacy events for MCH following the withdrawal of SURE-P in Nigeria.

Twenty-two in-depth interviews (IDI) were conducted with representatives of various advocacy groups who were selected during the document review as being involved in MCH in Nigeria. They included advocacy groups of civil society organizations (CSOs), development partners, nongovernmental organizations (NGOs), professional groups, media, donor agencies, academia and researchers.

Selection of participants was purposive [32], as often used in qualitative research to identify a group of people who have unique characteristics or are in similar conditions with the event being studied [33]. The sampling frame comprised national and subnational (states) actors as groups working at the national level, and those operating in one state (Anambra State) were selected. Advocacy events in the whole country following the withdrawal of SURE-P were mapped, involving the national and state levels. This provided us with people with different experiences that will be of interest to us. Participants were selected to reflect diversity in groups, their occupation and professional background.

### Data collection

IDIs with 22 participants were conducted using a pretested semi-structured interview guide. The guide explored the identification of the contextual issues in which the case was situated, identifying the policy advocates, coalition formation, roles, core beliefs, the context of MCH in Nigeria, outcome of advocacy and factors enabling or constraining coalition formation. Interview appointments were sought by telephone or personal visits. Interviews were conducted in participants’ offices. Interviews lasted an average of 60 minutes, were conducted in English, audiotaped with the consent of the respondent and transcribed verbatim, and transcripts sent back to participants to confirm the accuracy of the transcript. Data were collected using a question guide pretested in Enugu State. The findings from the pretest were used to review and refine the question guide.

### Data analysis

The primary unit of analysis within an ACF is the policy subsystem, which consists of those actors from a variety of public and private organizations who are perceived to be concerned with the poor MCH indices in Nigeria and seek to address it through a change in policy [34]. This framework can help identify people who share the same goals and establish means to maintain their political gains [20].

The document review was analysed using the manual content analysis method, while qualitative data audiotapes were transcribed verbatim, anonymized, double-coded in MS Word using colour-coded highlights and analysed using manual thematic and framework analysis. For the analysis, we organized the data, categorized them, created themes and patterns using the subheadings of the interview guides, identified emerging issues and searched for their explanations as suggested by Marshall and Rossman [35]. The coding was guided by the principles of “comparative analysis” [36], where coded elements were compared under the categories and patterns were identified. For quality assurance and rigour, two persons were paired, and each provided input on the coding templates. Thus there was transparency in the data analysis; it was validated, reliable and had members check the coding [36]. Findings were supplemented and validated with a literature review.

## Results

The results presented here were extracted from the documents reviewed and IDIs conducted. Table 1 shows the profile of the respondents.

**Table 1:** Profile of respondents

Participant/respondent codes	Location	Total	Male	Female
Policy-makers (national/subnational levels; P1, 2, 3, 4)	FCT/Anambra	4	2	2
Development partners	Abuja	1	1	
Coalition groups (D1, 2, 3)	Abuja	3	2	1
CSOs (C1, 2, 3)	Abuja and Anambra	3	1	2
NGOs (N1, 2, 3)	Abuja and Anambra	3	2	1
Professional groups (G1, 2)	Anambra and national	2	1	1
Media (M1, 2)	Abuja and Anambra	2	1	1
Academia/researcher (R1, 2)	Abuja and Anambra	2	1	1
Advocacy influencers (A1, 2)	Abuja and Anambra	2	1	1

The documents reviewed included reports of advocacy activities of some professional groups, CSOs, media documents, reports from coalition groups activities, reports of advocacy research conducted, national reports and policy analysis documents.

### Chronological description of events in MNCH in Nigeria (2005–2015)

MCH in Nigeria has experienced many national interventions and policy changes in recent times, due to the quest to combat the poor MCH indices. The interventions are as follows: The global Partnership for Maternal, Newborn and Child Health (PMNCH) was initiated in 2006 but was later changed to the Integrated Neonatal, Maternal and Child Health (INMCH) in 2007 and continued until 2014. Then the policy of free MCH services was initiated by the federal government in 2009 and implemented until 2015, and the Midwives Service Scheme (MSS) was established in 2009 and implemented until 2012. There was also the SURE-P Maternal and Child Health (SURE-P MCH) project, which was implemented in January 2012 and suddenly withdrawn in April 2015.

## Policy subsystem

### Key characteristics

Participants perceived that poor MCH indices drive the formation of different coalitions as seen in Table 1. These actors are either homogeneous (of the same profession) or heterogeneous (combining various professions) as shown in Table 1. These differences have led to different types of interorganizational arrangements. One of the respondents explained, “In some groups, membership is diverse with various levels of leadership, sometimes donors and government officials are invited to provide information and clarity for engagements” (CSO member)*.*

### Policy actors

The identified actors were primarily CSOs, NGOs, private sector groups, media and implementation partners. Others included government workers, donor agencies and health professional associations. They operate at various levels of the government (national and state levels). We identified that homogeneous and heterogeneous groups both have their advantages and disadvantages. The homogeneous groups tend to engage in greater collaboration since they belong to the same professional background, with a shared mission and a good network. As one respondent stated, “Being colleagues made it easier for us to harmonize our actions and being a respectable profession in the country, with the ability to help give ideas and work towards the reduction of this maternal and child death” (health professional group leader).

On the other hand, other groups enjoyed having members of various professions in their coalition groups (Table 2). This, they said, was due to increased access to information sharing, increased access to resources, heightened accountability and improved problem-solving, bringing in resources and shared ideas from a diverse array of persons as instrumental to their achieved success. As one participant noted, “We tap into the knowledge of some of us who are experts in that area, plus we also have members of the media in our group who give us free publicity, these helped us achieve our aim as a group” (NGO member).

**Table 2:** Distribution of advocacy coalition actors

Advocacy groups	Homogeneous	Heterogeneous	Operating at national level	Operating at state level
Coalition of CSOs (Partnership for Advocacy in Child and Family Health – PACFAH)	**√**		**√**	
Coalition of media houses (Champions for Maternal Health)	√			**√**
Coalition of development partners (Development Partners Group)	**√**		**√**	
Coalition of specific professional groups (Society of Gynaecology and Obstetrics of Nigeria – SOGON)	**√**		**√**	
Coalition of implementers		**√**		**√**
State-level coalition for accountability		**√**		**√**
Health Sector Reform Coalition (HSRC)		**√**	**√**	
Coalition for Maternal Newborn Child and Adolescent Health Accountability in Nigeria (C4MAN)		**√**	**√**	
Civil Society Scaling-Up Nutrition in Nigeria (CS-SUNN)		**√**	**√**	
Health Reform Foundation of Nigeria (HERFON)		**√**	**√**	**√**
State-led Accountability Mechanism for MNCH (SLAM)		**√**		**√**

### Perceived belief correspondence

Findings show that beyond a common meeting point for coalition/collaboration of the subsystem lie the core beliefs that drive the agenda of interest. Of the three-tiered belief system, namely deep core, policy core and secondary beliefs, for the ACF, the emphasis is on the policy core beliefs, which includes the policy goals of whether and how a government should or should not act in relation to a problem or concern. This policy core belief acts as the binder of coalitions. In this study, we used perceived belief correspondence which ascertained how important the policy subsystem is to the policy actors and their attachments to it. This was done by ascertaining the intensity of the belief and its importance of to their personal and professional goals, as illustrated by one of the participants: “All of us in this group are concerned and passionate about the health of mothers and children as our profession deals with saving them, we find it very difficult to understand why the government cannot put basic things in place to ensure their safety, especially during childbirth” (professional group leader). Then another said, “… first is the passion that drives the coalition, the second is the ability and the capacity of the coalition and then the unity of purpose. They must have a common vision and believe strongly in it to be able to achieve any result as a coalition as seen in our group” (CSO leader).

The subsystem is driven by their belief on the need to advance the MCH agenda and the desire to see the need reflected in reprioritization of MCH on the political agenda. The perceived deep core belief driving the MCH agenda is the right of an individual to health and their desire to protect the lives of so many children and mothers. This was described by respondents as their motivator.

### Coordination

Findings show that coalitions’ perceived beliefs were operating at three different levels. Those of the homogeneous groups were mostly deep core belief (the human rights angle of better health for mothers and children), and it was recognized that this enabled them to work together since they were like-minded, which drives better coordination of the group. The heterogeneous groups were perceived to possess all three levels of beliefs—some possessing the deep core belief, others possessing the policy core belief (these were convinced to join due to evidence presented by the deep core belief group), and yet others possessing the secondary belief, who were easier to convince to join the MCH advocacy. This also affected the actions of these groups, as they were difficult to coordinate and implement their plans, as was noted: “…you know in our group, there were few of us that really wanted this, but we can’t do it alone, we had to try and meet stakeholders to join us, but some were very difficult to convince and this affected us as those people were not really committed” (member of primary health centre [PHC] advocacy group).

### Group formation and strategies for engagement

Different strategies for initial engagement and processes of group formation were identified. The presence of an external factor usually triggers the formation of coalitions. In some instances, concerned individuals with identified core beliefs engage with key foundation members, to develop a strategy to reach some advocacy champions. They subsequently present their ideas to the larger group for group buy-in, thus forming a coalition. In other instances, some NGOs identify and engage policy influencers, like the wives of the governors (popularly called the first ladies), celebrities and known philanthropists, on the need for a change in MCH conditions in the country, and some of them joined the coalition. There were also instances where groups of researchers in MCH received grants to train the media on how to promote MCH issues in the country. Thus, in this instance, the researchers started by inviting the media groups and training them, thereby forming a coalition of media for MCH in Nigeria. This group is perceived to have achieved a great deal for the MCH sector in the country, as noted by one of the respondents: “A year later after our training as media coalition group, we came back and reported changes that we’ve seen in our various states. So, some of them for instance, have made their states buy into specific portions of things like primary health care under one roof. Some of them didn’t even have boards for primary health care, but they do now. Other states started a free maternal and child health programme” (member of media coalition group).

At inception, coalitions identify relevant stakeholders and powerful entry points, they develop their clearly stated objectives, map out a clear structure and administrative arrangement, then prepare evidence as a tool for advocacy and information campaigns. This evidence will highlight the enormity of the problem. They also identify a policy window of opportunity, engage similar or diverse team members and share tasks commensurate with the task at hand. They then identify policy influencers to facilitate achievement of set goals and involve the media to disseminate the information and shape the public’s view of the agenda. A respondent stated, “We were initially just few of us, then we identified some key stakeholders and approached them with our vision and the opportunity on ground invited the media too for coverage and visibility” (member of PHC coalition).

In all the advocacy coalition groups, we identified strong collaborative coordination as all coalition actors agreed upon and acknowledged their activities, such as implementation of their plans and sharing of their resources towards the achievement of the group’s common goal.

### Factors that enable group effectiveness/resources

Respondents mentioned factors that enabled group effectiveness and stability over time, which included clearly stated specific objectives, clarity of roles and tasks, links with powerful individuals and influencers, access to good-quality evidence, and funding to enable independent engagement. Other factors included the ability to attract more funds as needed, continuous capacity building to sustain their action, improved communication and accountability among members, group independence from government or donor, and a total focus on the agenda of interest. Most of these factors cut across all the groups. A respondent said, “Some of our members were only responsible for scouting and getting funds, this was needed for us to implement our plans and even send our members for proper training” (member of an implementation coalition).

Other key enablers included the existence of an enabling environment, existence of political will as seen by the willingness of the government to engage in discussions with advocates, global commitments which the country had committed to, commitment from the stakeholders to press for achievement of common goals, availability of funds, access to evidence and access to powerful policy influencers.

### Constraints and challenges

Some constraining factors that delay the policy process were noted as follows:

- Lack of proper coordination of the actors advocating for the same course. Respondents noted that different groups were seen to advocate to the same persons for the same course, and politicians reported that it was very distracting and consequently divided or diminished their attention to that issue.
- Strife for credits was noted as a vital obstacle. Respondents described this as situations in which some members of the coalition who did not contribute to the process (or those who provided more funding) were seen to quickly make claims to credit as a result of membership. A respondent stated, “Sharing achievements became a challenge as individual organizations sometime claim the success especially where they see themselves as having contributed more funding towards the achievement of milestones” (member of NGO).
- Restriction of the funding principles by some funding organizations; for instance, respondents noted that some global donors did not give room for lobbying, and hence any activity the coalition undertook under the support of such donors tended to have this limitation.
- Dominance of groups, projects and implementation partners by international NGOs over the indigenous members due to capacity and funding leads to lack of group integration, ownership and sustainability.
- Power conflict and struggle for recognition, roles and position amongst members was also highlighted as a major constraint to achieving proper group integration and group goals. As one respondent stated, “Conflicts always arise due to lack of cohesion and poor understanding among group partners” (policy implementers).
- The ability to converge group members for meetings is also a challenge, and without regular meetings they cannot achieve much. This was noted by a respondent: “… Some of the facilitators to advocacy activities are willingness of advocates to meet and agree on a common issue and work together, which is not easy at all. The main thing is being able to manage human beings and work in harmony. The second thing is to be punctual, have funds to work with and have good influencers to go with the group. Having the real facts and figures to go with the advocacy visits is another enabler to good advocacy” (member of development partner).
- Funding was seen as a source of challenge, especially at the initial stage of group coalition.
- Commercialization of advocacy activities by some group members was also highlighted as a constraint.

## External factors/triggers of coalition formation

Findings clearly show external events that drew attention to the MCH agenda and triggered the formation of MCH coalitions. The sudden withdrawal of SURE-P due to changes in the political environment, specifically a change in government that led to abandonment of the MCH programme instituted by the outgoing government, was seen as the trigger. As was noted, “Immediately the government stopped funding that SURE-P programme, many people reacted and that was the beginning of increased death of mothers and children, so we had to act fast” (member of state-level coalition).

International ranking that placed the country as the third worst country with regard to maternal mortality was also identified as an external event. This ranking drew international attention towards the country, and also led to increased political commitment by the government in a bid to please the international community. The poor MCH indices—though a stable parameter, because this has always been the case—can also be seen as a trigger. The much higher records of MCH indices in Nigeria in the prevailing year (814/100 000 live births) by the Development partners (DPs) in 2015, compared to the previous Nigeria Demographic and Health Survey (NDHS) 2013 (576/100 000 live births), which was the initial generally accepted figure, was also a source of a trigger for the subsystem.

We also identified the interference of global policies and other non-health polices, such as financial bills, on the implementation of national policies and interventions. These external forces influenced advocacy coalition groups to advocate for better lives for mothers and children.

### Context

There were contextual cultural and socioeconomic factors which have fuelled the poor MCH issues. These include inadequate funding, delayed release of budgetary allocation, poorly equipped health facilities, shortage of skilled staff, donor dependency, and fragmentation of programmes and health initiatives. It was noted, “In Nigeria, it is difficult to get money for MCH and the small one budgeted for this is hardly released and there are no qualified hands in the PHCs. This has been so for long” (member of the accountability coalition). Other factors identified included few empowered females, ignorance, poor health-seeking behaviour, low immunization uptake, poor access to health facilities, poverty and delivery by unskilled staff. The periodic change in political administrations without continuity in implementation of on-going projects was perceived as a norm in Nigeria. All these prevail in the Nigerian context and affect the MCH subsystem. They contribute to the MCH outcome as seen.

### The effects of advocacy groups on policy change

The role of coalitions in sustaining the prioritization of MCH issues on the political agenda was highlighted as critical to meaningful achievement of outcomes and introduction of interventions that are beneficial to mothers and children. These groups have been able to ensure, to a certain extent, the implementation of relevant policies, increased allocation and release of funds to MCH issues, strengthening of information and data management, and improvement in human resources for health such as engagement with health professional associations during industrial actions, amongst others. The advocacy by various coalitions has also led to improved efficiency and effectiveness in the entire management of MCH programmes and improved integration of processes across the various components of the MCH system. In the words of one respondent, “Various advocacy coalition groups were found to be instrumental in the enactment of different policies/programmes formulated to advance MCH in the country. Respondents highlight records of achievements of such coalitions in enactment of MCH policies—NSHDP [National Strategic Health Development Plan]; funding commitment; legal backing (policy commitment) and government ownership; increased funding for MCH; revitalization of PHCs; Human Resource for Health intervention—CHIPS [Community Health Influencers, Promoters and Services]” (leader of CSOs).

Yet another stated, “Now, we have the GFF [Global Financing Facility] funding that has come up, the GFF is talking about the RMNCAH [reproductive, maternal, neonatal, child and adolescent health] in the country which is part of the holistic plan of the country. You know we have the National Strategic Development Plan, too, that has been developed, which most states now have their own. Those also actually outlined the key issues of which MCH is a very vital one. There is issue of the CHIPS—the community health initiative programme, the strategic initiative of the federal government to ensure that the issues of MCH is receiving the proper attention” (member of CSO).

Another respondent further noted, “The issue of the revitalization of one thousand PHCs per ward is actually to bring health closer. The PHC will be able to provide the first client care and this will reduce maternal and child deaths. We achieved all these through advocacies done by groups” (member of CSO).

These coalition groups advocate for the MCH agenda through health system strengthening reforms and initiatives that are inclusive, for sustainability. They initiate action by generating evidence. These could be presented as articles, policy briefs and media briefs, press conferences and press releases, among others. They hold regular meetings and plan advocacy visits to mount pressure on government and policy-makers, armed with evidence. Groups described as most active in pushing the MCH agenda are the coalition of CSOs, media and professional organizations. A respondent noted that, “We act by engaging the government strategically, people like the minister of health, minister of national planning and minister of finance, as well as the policy-makers. We discourse with them and keep pushing them till we achieve what we want” (leader of CSOs).

Yet another respondent stated, “Our actions now led to the review of the State Primary Health Care Development Agency, you know, it was NPHCDA [National Primary Health Care Development Agency] before, so that process now led to that policy and it was now backed up with the National Health Act in terms of monetary release, talking about the provisions, recognizing and giving it that legal backing to that institution and government ownership” (member state level coalition).

The specific activities carried out by coalition members were also identified, as shown in the Table 3. They shared the tasks such that their members worked on the various stages of the policy process, thus raising awareness, engaging stakeholders to influence policy processes and supervising all aspects of policy implementations.

**Table 3:** Activities carried out by members of advocacy coalitions at the four policy stages to ensure MNCH prioritization in Nigeria

Policy processes	Activities carried out by advocacy coalitions to ensure MNCH prioritization in Nigeria
Agenda setting	They raised awareness, highlighted the problem, attracted public and government attention, identified stakeholders to work with and policy window to act
Policy formulation	Engaging policy influencers, providing technical expertise
Facilitating implementation	Media involvement: sharing tasks to cover all areas of implementation progress according to members’ expertise
Policy evaluation	Budget reviews and feedback to government

### Interpretive analysis applying the ACF lens

These findings show the presence of actors in the policy subsystem who share policy core beliefs and are coordinated. They are perceived to be of the dominant subtype, and some perceived cooperative subtypes also exist. It is important to note that the ideal type of coalition may not be the best and may not be the preferred subtype, as their composition and behaviour depend on the context and the course for which they were formed. In this study, the ideal subtype was seen, and it helped the course of the coalitions in achieving set goals. Although it is known that beliefs may shape coordination, this is not true in all instances. In this study, we saw that the coordination was mostly among allies; they were strong coalitions, and the risk involved was very low, as there was no highly sensitive information or data involved with minimally risky joint activities. This study prefers to call these coalitions belief coalitions due to the indicators used, in order to avoid making claims. Table 4 provides a summary of the analysis.

**Table 4:** Summary of application of ACF to MCH policy advocacy in Nigeria

Territorial scope	Sub-territorial scope	Subsystem	Policy core beliefs	Advocacy coalitions	External shock	Policy brokers	Perceived outcome of advocacy events
Nigeria (national and state levels)	MCH policy sector in Nigeria	Actors: CSOs, NGOs, private sector, media, implementation partners, government workers, donor agencies, health professional associations	Poor MCH. Right of an individual to life and health. The desire to protect the lives of so many children and mothers	Coalition of CSOs, coalition of development partners, coalition of specific professional groups, coalition of media houses, coalition of implementers, coalition of state-level coalition for accountability, health sector reform coalition (HSRC)	High maternal mortality result published by development partners. Sudden withdrawal of the free MCH programme following a change in government	Policy decision-makers, mainly the government at the national and state levels	Free MCH in some states, enactment of MCH policies—NSHDP, increased funding for MCH; revitalization of PHCs; human resources for health intervention increased accountability in MCH programmes Introduction of new interventions country-wide e.g. Basic Health Care Provision Fund (BHCPF), CHIPS, reduced MCH indices by 2018, legal backing of MCH activities, government ownership of programmes

## Discussion

The competing health needs of diverse populations and ever-reducing resources available to support these needs often serve as the drive for the initiation of advocacy to improve health outcomes. In this study, the poor MCH indices and the withdrawal of the SURE-P MCH programme led to various advocacy events. As noted elsewhere, such events were carried out by actors who were motivated not just by a logic of consequences but by a logic of appropriateness [37]. Some of these advocates in Nigeria formed coalitions that possessed the characteristics that had been identified internationally by authors as being enabling factors for success, including gaining status, access, resources and diversity in groups [38, 39].

The coalitions seen in this study are loose collections of alliances made up of committed individuals and institutional policy actors with dense interorganizational and interpersonal ties working together to influence policy. They develop a common agenda, shared priorities for action and collaborative advocacy initiatives to ensure governmental accountability to health commitments. Their common agenda was found to be critical to building a movement of actors advocating for better access to health services, information and funding across Nigeria, as seen in other studies [40]. Globally, these coalitions are otherwise called networks and have been instrumental in the ascent of health issues to the political agenda of global health [41, 42, 43]. This formation of advocacy coalitions is like those identified by Sabatier and Jenkins [14]. It was one of the ways that enabled effective advocacy, probably because actors enjoyed the collaborative benefits of networking by engaging in collective actions that increased their visibility, and thus they were able to attract the much-needed attention that made their voices heard, as seen in this study. This corroborates the findings of Matti, who examined the rationale determining advocacy coalitions [44]. In this study, strong collaborative coordination exists within advocacy coalition groups, mostly driven by their primary core beliefs, although studies have shown that secondary beliefs can also drive coalition [45, 46].

As stated in the ACF hypothesis, policy changes do not occur without a trigger, otherwise known as an external shock [14, 47]. External shock has been defined as broad changes in socioeconomic conditions, public opinion, governing coalitions and changes in other subsystems. In this study, the external shock identified was the sudden withdrawal of the SURE-P programme due to a change in governance following the election of a new political party who did not want to continue the activities of the last government. Also, the increased maternal and child death indices from the development partners equipped the advocacy coalition groups with substantive evidence and led to increased awareness of the MCH issue in the country. These led to increased political will, as the government was eager to act in a positive way to please the international community. This enhanced the achievement of advocacy outcomes, as shown by this study.

Our findings correspond with existing literature, as shown from a study that achieved policy change following an external shock in Sweden, though they emphasized the need for further research to uncover the micro-level processes at work when policies change following external shocks [46]. This is contrary to the findings of the study from Mintrom and Vergari [47] and Ameringer [48], who observed that not all external shocks lead to policy change, and not all policy changes result from external shocks [49]. But the ACF by Sabatier and Weible [13] stressed that external shocks have other very important effects apart from leading to a policy change, such as leading to shifts in agendas, and focusing public attention on the issue, thus attracting the attention of key decision-makers. These effects were also identified in this study. The withdrawal of the programme and the activities carried out by the coalition groups highlighted the need for reprioritization of MCH, and this was achieved. This is especially necessary if the country hopes to achieve the health-related SDGs as stated by WHO [50].

External shock also causes the redistribution of resources within a policy subsystem. Policy change in ACF is due to competition within the subsystem as well as to external events. The competition within the subsystem usually involves a substantial conflict in goals and important technical disputes [13]. Each coalition therefore mobilizes its resources to achieve belief supremacy. Such resources include formal legal authority, public opinion, information dissemination, financial resources and skilful leadership [19].

In this study, though the coalitions are all working towards the achievement of improved MCH care for the citizens, they still operate at various levels of the policy process and still favourably compete amongst themselves (the coalitions) and amongst the actors in the coalition groups. Each group is struggling to achieve dominance within the MCH realm, and thus various coalition groups were seen mobilizing resources and attracting influential personalities and policy champions to their groups. The power of the actors in a group largely determined their ability to gain access to policy-makers and expedite the achievement of their set goals, as shown in other advocacy studies in Nigeria [51], while within the groups, the findings revealed conflicting priorities amongst coalition members. This stemmed from struggles for position, recognition and power, though they appear to take collective actions to protect their coalitions. This is similar to the findings in WHO’s networking and coalition-building for health advocacy [52], and in Uganda, where civil societies have advocated for sexual and reproductive health rights [53].

It is also reported that the formation of coalitions, the mobilization of its members and resources, information dissemination and their overall advocacy will to a great extent depend on the existing political system, and that a democratic and stable political system is vital for these processes to be accomplished. This was identified as the major bone of contention concerning the applicability of the ACF in countries outside the United States that may have different or not so stable political systems [14]. But in this study, though the ruling political parties have been changing, there has been some stability with regard to the change of governance following the political cycle in Nigeria. A previous study also applied ACF in policy changes in Nigeria [19].

In the ACF, actors in policy subsystems are mostly driven by their beliefs and their desire to see their beliefs reflected in the various MCH policy processes. This was seen as the core motivator for coalition formation in this study, especially for the homogeneous groups, and it explains the behaviour of advocacy coalitions. Actors have also been known to influence health policy formulation through the meaning they attach to their experiences and beliefs [32]; these have also been instrumental to health issue prioritization as seen in other countries [23, 54] and also in non-health issues [55]. This study also shows that their perceived policy belief is the driving mechanism behind group coordination, especially for the homogeneous groups, as seen in other studies [18, 44]. This confirms our working hypothesis and, as explained by Weible et al. [56], may result from the wide variety of ways in which beliefs are conceptualized and measured [57].

### Implications for policy and practice

From our research, some implications for policy and practice concerning the formation of advocacy coalitions and their effects on policy change are evident: Achieving coalition group-set goals should be key to coalition formation, and avenues for achieving this should be sought. To this end, a clear vision, mission and goals should be made known to members at an early stage, and resources apportioned and channelled towards achieving the set goals. Also, policies should be written with an understanding of the contexts in which they are to be implemented, to allow for flexibility at the local level. Advocates should also find ways to attract and engage the right members that will lobby policy-makers early in their group formation, as their success may depend on this. Advocacy coalition groups should plan to evaluate the impact of their efforts early and often, as such information could lead to continuous improvement in the implementation of reform initiatives and ultimately to greater success.

### Implications for research

This study has shown that the ACF can be widely applied in various settings; however, researchers should identify the indicators that will help them measure the variables for identifying coalition formation before applying ACF in research, as this will help them classify the coalition types and subtypes, thereby contributing meaningfully towards discussions around the ACF. Also, future research should consider a combined qualitative and quantitative study approach to enable the systematic collection of data and measurement of variables.

### Limitations

A key limitation of case study design, and one that is applicable to this study, is that given the emphasis on the in-depth nature of the results, the research cannot be feasibly carried out on a large scale and so may not be used to make conclusions in other settings. However, the aim was not to generalize findings but to contribute to the growing literature of advocacy in the first instance, providing new knowledge on MCH advocacy in the study setting, and within this, there may be transferable concepts for other settings.

This study did not measure deep core beliefs, or empirically ascertain the content and structure of belief systems. It was recommended that this not be undertaken for any ACF studies, but it will be important for those studies seeking to describe and explain aspects of different levels of ACF belief systems and test hypotheses about them, which we are not doing in this study.

It is also difficult to attribute the gains in reprioritization of MCH in Nigeria to the activities of the advocacy coalition groups alone, as there are other contextual issues that played out at about the same time. Also, the views here are solely those of the study participants, and the small number of participants interviewed makes it difficult to generalize the findings of this study.

## Conclusion

Our findings add to an increasing body of evidence that the use of ACF is beneficial in exploring how advocacy coalitions are formed and their contributions towards the prioritization of MNCH issues on the political agenda, as well as the release of resources commensurate with the gravity of the problem. The coalitions have brought about a positive shift in decision-makers’ attitudes and increased policy support for MCH issues in Nigeria, especially following the suspension of the free MCH programme.

Formation of advocacy coalitions took different approaches, but all stemmed from recognizing the poor MCH situation in the country, as well as health system problems which constrained MCH policy processes. The various mechanisms of collaborative coalition formation possess key characteristics which include formation of groups, thus combining power, aligning their objectives/shared purpose, coordinating their activities and sharing their resources to achieve set goals. Though each type of coalition has its benefits, the homogeneous groups were at the forefront in achieving expected outcomes due to their value-oriented perceived core belief, coordination networks and ability to easily trade off and prioritize to achieve set goals.

This study proposes that for advocacy coalitions to be effective, members need to have a good understanding of their group’s goals, vision and mission, with strong core beliefs and passion towards nurturing the coalition for its stability and sustainability. Future policy advocates should take advantage of coalitions as powerful tools, but take note of their identified challenges and context-specific issues.

## Data Availability

The datasets used and/or analysed during the current study are available from the corresponding author on reasonable request.

## Abbreviations

ACF: Advocacy coalition framework
ANC: Antenatal clinic
BHCPF: Basic Health Care Provision Fund
CHIPS: Community Health Influencers, Promoters and Services
CSO: Civil society organization
IDI: In-depth interview
MCH: Maternal and child health
MOF: Ministry of Finance
MOH: Ministry of Health
NGO: Nongovernmental organization
NSHDP II: National Strategic Health Development Plan Framework II
PHC: Primary health centres
SURE-P: Subsidy Reinvestment and Empowerment Programme
UNICEF: United Nations Children’s Fund
